# Action potential clamp characterization of the S631A hERG mutation associated with short QT syndrome

**DOI:** 10.14814/phy2.13845

**Published:** 2018-09-02

**Authors:** Andrew Butler, Yihong Zhang, Alan G. Stuart, Christopher E. Dempsey, Jules C. Hancox

**Affiliations:** ^1^ School of Physiology Pharmacology and Neuroscience Medical Sciences Building University Walk Bristol United Kingdom; ^2^ Bristol Heart Institute University of Bristol Bristol United Kingdom; ^3^ School of Biochemistry Medical Sciences Building, University Walk Bristol United Kingdom

**Keywords:** Arrhythmia, atrial fibrillation, hERG, KCNH2, quinidine, S631A, short QT syndrome

## Abstract

The hERG potassium channel is critical to normal repolarization of cardiac action potentials (APs) and loss‐ and gain‐of‐function *hERG* mutations are associated, respectively, with long and short QT syndromes, pathological conditions that can lead to arrhythmias and sudden death. hERG current (*I*_h_ _ERG_) exhibits uniquely fast inactivation involving conformational changes to the channel pore. The S631A hERG pore mutation was originally engineered to interrogate hERG channel inactivation, but has very recently been found in a family with short QT syndrome (SQTS). Accordingly, this study characterized the effects of the S631A mutation on *I*_h_ _ERG_ profile during ventricular, atrial, and Purkinje fiber (PF) AP waveforms, using patch clamp recording from hERG expressing HEK 293 cells at 37°C. Under conventional voltage clamp, the current–voltage (*I*–*V*) relation for *I*_h_ _ERG_ exhibited a marked right‐ward shift in the region of negative slope at positive membrane potentials. Under ventricular AP clamp, the S631A mutation resulted in augmented *I*_h_ _ERG_, which also peaked much earlier during the AP plateau than did wild‐type (WT) *I*_h_ _ERG_. Instantaneous *I*–*V* relations showed a marked positive shift in peak repolarizing current during the ventricular AP in the S631A setting, while the instantaneous conductance‐voltage relation showed an earlier and more sustained rise in S631A compared to WT *I*_h_ _ERG_ conductance during ventricular repolarization. Experiments with atrial and PF APs in each case also showed augmented and positively shifted *I*_h_ _ERG_ in the S631A setting, indicating that the S631A mutation is likely to accelerate repolarization in all three cardiac regions. Ventricular AP clamp experiments showed retained effectiveness of the class Ia antiarrhythmic drug quinidine (1 *μ*mol/L) against S631A *I*_h_ _ERG_. Quinidine is thus likely to be effective in reducing excessively fast repolarization in SQTS resulting from the S631A hERG mutation.

## Introduction

The process of ventricular repolarization in the heart involves the combined activities of a number of potassium (K^+^) channels (Tamargo et al. 2004). Among these, the rapid delayed rectifier K^+^ current, *I*_Kr_, is of particular note, due to its association with both congenital and acquired arrhythmias (Modell and Lehmann 2006; Sanguinetti and Tristani‐Firouzi 2006; Hancox et al. 2008; Hancox and Stuart 2018). Channels mediating *I*_Kr_ are encoded by *human Ether‐a‐go‐go Related Gene* (*hERG*; alternative nomenclature *KCNH2* (Sanguinetti et al. 1995; Trudeau et al. 1995)) and are characterized by a uniquely rapid voltage‐dependent inactivation process that limits *I*_Kr_/hERG current (*I*_hERG_) at positive membrane potentials (Sanguinetti et al. 1995; Trudeau et al. 1995; Smith et al. 1996). As a result, *I*_Kr_ normally contributes little to repolarization early during the ventricular action potential (AP), but its contribution increases progressively, peaking just before the terminal repolarization phase of the AP, which is mediated by inwardly rectifying K^+^ current, *I*_K1_ (Ibarra et al. 1991; Hancox et al. 1998a; Zhou et al. 1998; Mitcheson and Hancox 1999). Inactivation gating of hERG channels is also important for some (typically high affinity) hERG blocking drugs to interact optimally with their binding site(s) on the hERG channel (Sanguinetti and Tristani‐Firouzi 2006; Hancox et al. 2008).

In 2000 a condition called Short QT Syndrome (SQTS), involving abbreviated ventricular repolarization and susceptibility to arrhythmia was first identified (Gussak et al. 2000). *hERG* was the first gene implicated as causing SQTS in 2004, when a missense mutation in the S5‐Pore linker region (N588K) of the hERG channel was found in SQTS families (Brugada et al. 2004). This mutation leads to a profound positive shift in the voltage dependence of inactivation of *I*_hERG_, with the consequence that little inactivation occurs over physiologically relevant membrane potentials and the size and timing of *I*_hERG_/*I*_Kr_ during ventricular APs is significantly altered (Cordeiro et al. 2005; McPate et al. 2005). The changes in kinetics of *I*_hERG_/*I*_Kr_ caused by this mutation result in AP and rate‐corrected QT (QT_c_) interval abbreviation and to shortening of the effective refractory period, which in turn lead to an increased susceptibility to re‐entrant arrhythmia (Adeniran et al. 2011). Since the N588K hERG mutation was linked to the SQTS, a number of other hERG mutations have been identified in SQTS patients, though until very recently none were found that have such a profound effect on the channel's inactivation process as does N588K (for a review see Hancox et al. 2018).

The S631A hERG mutation has long been used as an experimental tool to explore the inactivation process of hERG and the effect of inactivation removal on drug binding. This serine residue is located in the outer mouth of the channel and Schoenherr and Heinemann (1996) mutated it to match the analogous (alanine) residue in non‐inactivating *eag*‐encoded channels, with the consequence that inactivation of the hERG channel was profoundly attenuated (Schoenherr and Heinemann 1996). A subsequent detailed characterization of S631A hERG channels expressed in *Xenopus* oocytes reported a >100 mV positive shift in half maximal inactivation (Zou et al. 1998), while limited AP voltage clamp data from this laboratory, using a modified ventricular AP command, showed a marked effect of the mutation on current timing during the AP (Hancox et al. 1998b). The S631A mutation was subsequently shown to affect differentially the inhibitory actions on hERG of the Class III antiarrhythmic drug dofetilide (which was greatly affected) and the Class Ia antiarrhythmic drug quinidine (which was little affected) by the loss of inactivation due to the mutation (Lees‐Miller et al. 2000). The mutation has subsequently been used widely in the study of drug binding.

Very recently, the S631A hERG mutation has been reported to occur in an SQTS family for the first time (Akdis et al. 2018). The mutation was found in a 6‐year‐old girl who was screened after the sudden death of a cousin. She, her father and sister were all reported to have abbreviated QT_c_ intervals (of 340 msec or less, with the index patient <320 msec). The index patient was asymptomatic until she experienced syncope a decade later and was fitted with an implantable defibrillator (ICD). She, her father and sister were found to possess the S631A mutation, while an asymptomatic brother did not. There was also a history of sudden death on the father's side of the family (Akdis et al. 2018). The index patient also expressed an *SCN10A* variant, but her father did not and her mother, who also had the *SCN10A* variant, did not have a short QT interval (Akdis et al. 2018). No in vitro characterization of S631A was conducted in that study, but from what is already known, the mutation's effects on repolarization are likely to be profound. The AP voltage clamp technique has been used to good effect in characterizing the effects of the N588K hERG mutation (Brugada et al. 2004; Cordeiro et al. 2005; McPate et al. 2005, 2009), including use to compare the effects of the mutation on *I*_hERG_ during different configuration AP waveforms from different cardiac regions (Cordeiro et al. 2005; McPate et al. 2009). The study that is the subject of this report was undertaken: (i) to provide comparative information on the effects of the S631A mutation on the profile of *I*_hERG_ during atrial, ventricular, and Purkinje fiber APs; (ii) to establish effectiveness of quinidine on S631A *I*_hERG_ under ventricular AP voltage clamp.

## Methods

### Cell culture and hERG expression

Human embryonic kidney cells (HEK 293; European Collection of Cell Cultures, Porton Down, UK) were used for all experiments. These were cultured at 37°C in Dulbecco's minimum essential medium with Glutamax‐1 and supplemented with 50 *μ*g mL^−1^ gentamycin and 10% fetal bovine serum (Gibco, Paisley, UK). As demonstrated previously, the attenuation of inactivation caused by the S631A mutation causes significantly larger *I*_hERG_ in mammalian cell expression (Hancox et al. 1998b; McPate et al. 2008). Consequently, a reduced volume of the mutated cDNA was transfected into the cells in this study to prevent excessively large currents and ensure accurate recordings. Transient transfection of 1 *μ*g WT or 0.5 *μ*g S631A hERG cDNA, as previously (Hancox et al. 1998b; McPate et al. 2008; Melgari et al. 2015) was performed using Lipofectamine 2000 as per the manufacturer's instructions (Invitrogen, Paisley, UK). 0.15 *μ*g of CD8 expression plasmid (in pIRES, donated by Dr I Baró, University of Nantes, France) was co‐transfected to use as a marker of expression, with successfully transfected cells being identified using Dynabeads^®^ (Invitrogen, Paisley, UK) as performed previously (Zhang et al. 2011; Du et al., 2014). Following transfection, cells were dissociated using trypsin‐EDTA (Sigma‐Aldrich, Gillingham, UK) and plated on to 13 mm glass coverslips in Petri dishes containing the same medium as used above to enable electrophysiological recordings.

### Solutions for electrophysiological recordings

Similar to previous studies (McPate et al. 2005, 2008, 2009; Zhang et al. 2011), normal Tyrode's solution containing (in mmol/L): 140 NaCl, 4 KCl, 2.5 CaCl_2_, 1 MgCl_2_, 10 Glucose, and 5 HEPES was titrated to pH of 7.45 with NaOH for superfusion over the cells in a recording chamber at 37 ± 1°C. Patch‐pipettes, fire polished to 2–4 MΩ, were filled with dialysis solution containing (in mol/L): 130 KCl, 1 MgCl_2_, 5 EGTA, 5 MgATP, and 10 HEPES, titrated to pH of 7.2 using KOH (McPate et al. 2005, 2008, 2009; Zhang et al. 2011). Quinidine powder (Quinidine gluconate salt; Sigma‐Aldrich, Gillingham, UK) was dissolved in Milli‐Q water to produce a 1 mmol/L stock solution, and this was subsequently diluted to 1 *μ*mol/L in normal Tyrode's solution to be used for experimentation.

### Experimental protocols and analysis

An Axopatch 200A amplifier (Axon Instruments, Foster City, CA, USA) and a CV201 head‐stage were used to make whole‐cell patch clamp recordings of membrane currents. All data were recorded via a Digidata 1440A interface (Molecular Devices, Sunnyvale, CA, USA) using a bandwidth of 2 kHz and a digitization rate of 10 kHz. At least 70% of the electrode series resistance could be compensated. Excel 2016 (Microsoft, Redmond, WA), Prism 7 (Graphpad Inc, La Jolla, CA, USA), Clampfit 10.2 (Axon Instruments) and Origin 2017 (OriginLab Corporation, Northampton, MA, USA) were all used for data analysis as appropriate.

The relevant voltage protocols used are detailed within the Results section. The action potential waveforms used in action potential voltage clamp (AP clamp) experiments have been employed in previous studies (McPate et al. 2005, 2008, 2009; El Harchi et al. 2010; Milnes et al. 2010; Zhang et al. 2011), and the currents elicited by these were corrected online for P/N leak subtraction using an interspersed P/4 protocol (McPate et al. 2005, 2008, 2009; El Harchi et al. 2010; Zhang et al. 2011). Successive APs were applied at a start‐to‐start interval of 1 sec (Figures 2, 3, 4) or 12 sec (Figure 5). Total charge carried during ventricular AP in the presence or absence of quinidine was determined by integrating currents using Origin 2017.

**Figure 1:**
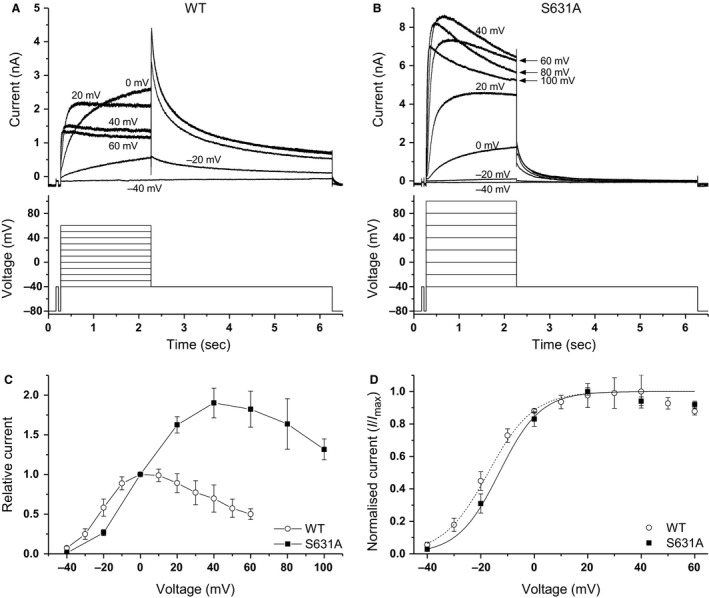
Current–voltage (*I*–*V*) relationship for WT and S631A hERG. (A, B) Representative current traces for WT (A) and S631A (B) hERG channels elicited by the protocols shown below. WT recordings were made using 10 mV voltage step increments between −40 mV and +60 mV, whereas S631A recordings used 20 mV increments between −40 mV and +100 mV in order to fully encapsulate the shift in current rectification. Selected traces are shown for clarity. (C) Mean *I*–*V* relations for end pulse WT (*n* = 7) and S631A (*n* = 5) *I*_hERG_, normalized to current at 0 mV. (D) Mean normalized tail current *I*–*V* relations for the same WT (*n* = 7) and S631A (*n* = 5) experiments shown in C. Currents were normalized to the peak current recorded during the protocol for each cell.

Voltage‐dependent activation was obtained from plots of normalized *I*_hERG_ tails against voltage (Fig. 1D) with a fit to the data with a Boltzmann equation of the form:

$$I=I_{\max}/\left(1+\exp\left(\left(V_{0.5}-V_{m}\right)/k\right)\right)$$

where the half‐maximal activation voltage (*V*_0.5_) was obtained by normalizing *I*_hERG_ tail current values (*I*), following differing voltage commands, to the maximal *I*_hERG_ tail value observed during the voltage protocol (*I*_max_), plotting the resulting values against corresponding command voltage (*V*_m_) and *k* is the slope factor describing *I*_hERG_ activation.

Fractional block of *I*_hERG_ by quinidine was calculated by the following equation:

$$\text{Fractional block}=1-(I_{\mathrm{hERG}}\left[\text{Drug}\right]/I_{\mathrm{hERG}}\left[\text{Control}\right])$$

where *I*_hERG_ [Drug] and *I*_hERG_ [Control] represent peak repolarizing currents or total current integral as appropriate; in the presence and absence of quinidine, respectively.

As in recent work from our laboratory (Helliwell et al. 2018), we used to the recent cryo‐EM open pore structure of hERG [PDB: 5VA1] (Wang and MacKinnon 2017) to show the structural context of the S631 residue. The structural figure (Fig. 6) was made using Pymol version 1.4 (Schroedinger, LLC, New York, NY, USA).

‘*n*’ values in the Results text and Figure Legends and that were used for statistics refer to numbers of individual cells from which observations were made. All data are presented as the mean ± standard error of the mean (SEM), and statistical comparison made using a Student's *t*‐test or one‐ or two‐way analysis of variance (ANOVA) and subsequent Bonferroni post hoc test. *P* values of less than 0.05 were considered significant.

## Results

### Effects of S631A mutation on *I*_hERG_ under conventional voltage clamp

In initial experiments, a conventional voltage clamp protocol (lower traces of Fig. 1A and B) was used to elicit *I*_hERG_ across a range of test potentials. For WT *I*_hERG_ a holding potential of −80 mV was followed by 2 sec test pulses to voltages between −40 mV and +60 mV, with subsequent repolarization to −40 mV and then a return to the holding potential (McPate et al. 2005; Zhang et al. 2011). As previously for N588K *I*_hERG_, (McPate et al. 2005; Zhang et al. 2011) this protocol was modified to include test voltages up to +100 mV in S631A‐hERG expressing cells in order to fully capture current rectification and the region of negative slope in the current–voltage (*I*–*V*) relation, that was positively shifted along the voltage axis. For the protocol used to study S631A *I*_hERG_, successive commands were separated by 20 mV rather than 10 mV increments, in order to increase the likelihood of successful completion of the protocol at highly positive voltages. For WT *I*_hERG_, current during the test pulse increased up to 0 mV and then declined at more positive voltages. Following test pulses to positive test potentials, resurgent tail currents were evident. For S631A *I*_hERG_, on the other hand, current increased progressively with increasing test potential magnitude up to +40 mV, at which point current started to decline. Additionally, for S631A hERG the tail currents on repolarization to −40 mV were smaller than *I*_hERG_ during the test pulse, across the entire range of voltages examined. Figure 1C shows the mean *I*–*V* relations for *I*_hERG_ (normalized to current at +0 mV for the two expression conditions (McPate et al. 2005)), showing that the region of negative slope in the *I*–*V* relation was shifted to more positive potentials for S631A. Figure 1D shows normalized *I*–*V* plots for WT and S631A *I*_hERG_ tails, fitted with a Boltzmann equation to establish the voltage dependence of activation. The half maximal voltage of activation was not significantly different between the WT (−16.8 ± 3.0 mV; *n* = 7) and S631A *I*_hERG_ (−14.4 ± 1.8 mV; *n* = 5; *P* > 0.05), and no change was seen in the slope (*k*) of the fitted activation relation (8.0 ± 1.5 mV for WT vs. 5.3 ± 1.0 mV for S631A; *P* > 0.05). These comparative properties of WT and S631A *I*_hERG_ are similar to those previously reported (e.g., (Schoenherr and Heinemann 1996; Zou et al. 1998; Hancox et al. 1998b; McPate et al. 2008). Positively shifted *I*_hERG_ inactivation has previously been well documented for the S631A mutation (e.g., Schoenherr and Heinemann 1996; Zou et al. 1998; McPate et al. 2008) and under the same measurement conditions as this study, previous work from this laboratory reported half maximal inactivation voltage for S631A *I*_hERG_ to be positively shifted by ~+88.5 mV (from −58.5 mV to + 30 mV; (McPate et al. 2008). As this voltage shift was recorded under similar measurement conditions to those used for this study, we did not repeat that experiment.

### Effects of S631A mutation on *I*_hERG_ elicited by ventricular AP voltage clamp

**Figure 2:**
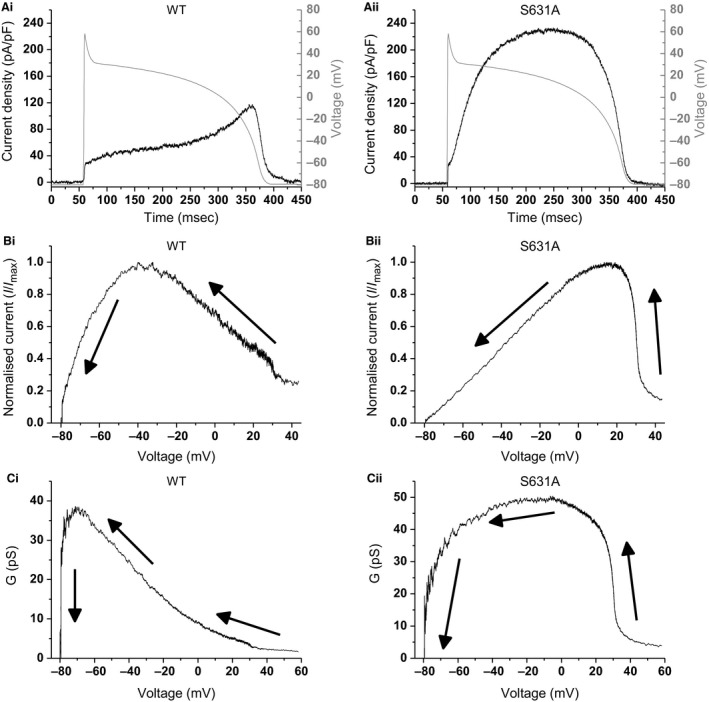
*I*_hERG_ during ventricular AP clamp. (A) Representative traces of WT (from a total of *n* = 6) and S631A *I*_hERG_ (from a total of *n* = 7) in response to a ventricular AP (overlaid in gray). (B) Representative plots of the instantaneous *I*–*V* relations during repolarization phase of the ventricular AP waveform. From 5 msec after the AP upstroke peak, currents were normalized to the maximal current recorded during the protocol. (C) Representative traces for the conductance–voltage (*G*–*V*) relationship. In (B, C) the arrows represent the direction of repolarization.

Figure 2A shows representative traces of WT and S631A *I*_hERG_ recorded under ventricular AP clamp. As expected (Hancox et al. 1998a; Zhou et al. 1998; Lu et al. 2001; McPate et al. 2005), there was relatively little WT *I*_hERG_ early during the imposed AP command, but this increased progressively throughout the AP plateau, peaking just before the steep terminal repolarization phase of the AP (Fig. 2Ai). The profile of S631A *I*_hERG_ differed, being larger, rising and peaking earlier during the AP plateau than for the WT channel, which gave rise to an inverted U‐shaped current profile (Fig. 2Aii cf (Hancox et al. 1998b; McPate et al. 2005)). Figure 2Bi and Bii show the instantaneous *I*–*V* plots for WT and S631A *I*_hERG_ during AP repolarization (direction of repolarization indicated by the arrows on the plots shown). For 6 cells, WT *I*_hERG_ was maximal at −32.2 ± 2.3 mV, whereas in 7 cells S631A *I*_hERG_ was maximal at +21.5 ± 2.6 mV (*P* < 0.0001 vs. WT). Thus, the S631A mutation significantly shifted the timing of *I*_hERG_ to peak earlier during the ventricular AP waveform. Figure 2Ci and Cii show the instantaneous *G*–*V* plots for WT and S631A *I*_hERG_, which take into account the instantaneous changes in driving force at each potentials as AP repolarization proceeds (Hancox et al. 1998a; Lu et al. 2001; McPate et al. 2005, 2009). In the WT condition, the instantaneous *G*–*V* relation increased progressively until relatively late during the ventricular AP command. In contrast, for S631A hERG the *G*–*V* relation rose much earlier during the AP plateau, reaching a maximum between ~0 and −30 mV and then progressively declining as the action potential progressed. Thus, S631A hERG contributed substantial outward conductance, earlier during the AP and over a larger voltage range than did WT hERG.

### Effects of S631A mutation on *I*_hERG_ during atrial and Purkinje fiber APs

**Figure 3:**
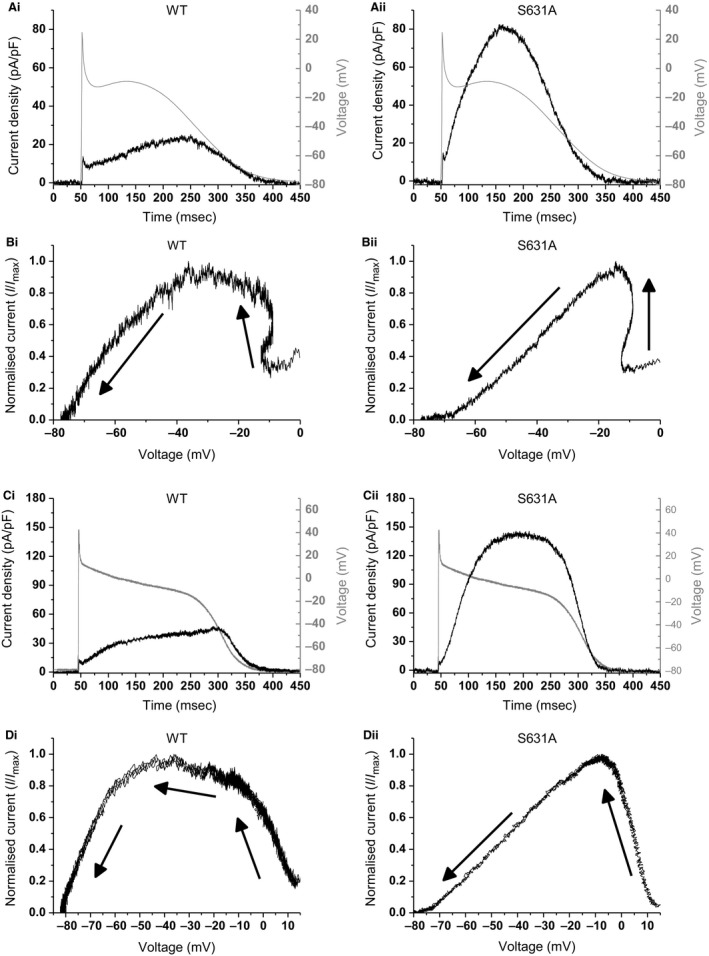
*I*_hERG_ during atrial and PF AP clamp. (A) Representative traces of WT (from a total of *n* = 6) and S631A (from a total of *n* = 6) *I*_hERG_ in response to an atrial AP (overlaid in gray). (B) Representative plots of the instantaneous *I*–*V* relations during the repolarization phase of the atrial AP command waveform. From 5 msec after the AP upstroke peak, currents were normalized to the maximal current recorded during the protocol. (C) Representative traces of WT (from a total of *n* = 6) and S631A (from a total of *n* = 6) *I*_hERG_ in response to Purkinje fiber AP (overlaid in gray). (D) Representative plots of the instantaneous *I*–*V* relations during the repolarization phase of a Purkinje fiber AP. From 5 msec after the AP upstroke peak, currents were normalized to the maximal current recorded during the protocol. In B and D the arrows represent the direction of repolarization.

Figure 3 shows the results of AP clamp measurements of WT and S631A *I*_hERG_ during atrial (Fig. 3A and B) and Purkinje fiber (PF) (Fig. 3C and D) AP waveforms. Due to the different profile of the atrial from the ventricular AP and, in particular, to a less positive plateau phase of the atrial AP, the profile of WT *I*_hERG_ during the AP differed from that in Figure 2, with a more modest increase in *I*_hERG_ throughout the repolarization phase and a relatively flat peak between −20 and −40 mV (Fig. 3Ai and Bi). The mean voltage at which WT *I*_hERG_ peaked during atrial AP repolarization was −25.7 ± 3.0 mV (*n* = 6). S631A *I*_hERG_ was increased in magnitude and slightly positive shifted compared to the WT channel (Fig. 3Aii and Bii), with peak current during repolarization occurring at −13.8 ± 0.7 mV (*n* = 6; *P* < 0.01). The Purkinje fiber (PF) AP used was intermediate between the atrial and ventricular APs in terms of plateau and the profile of WT *I*_hERG_ was more similar to that during the ventricular than during the atrial AP command (Fig. 3Ci and Di). Similar to the ventricular AP command, WT *I*_hERG_ peaked at −38.1 ± 2.0 mV (*n* = 6). S631A *I*_hERG_ was again augmented compared to WT *I*_hERG_ and the voltage at which maximal *I*_hERG_ occurred was positively shifted to −3.9 ± 1.8 mV (*n* = 6; *P* < 0.0001; Fig. 3Cii and Dii).

**Figure 4:**
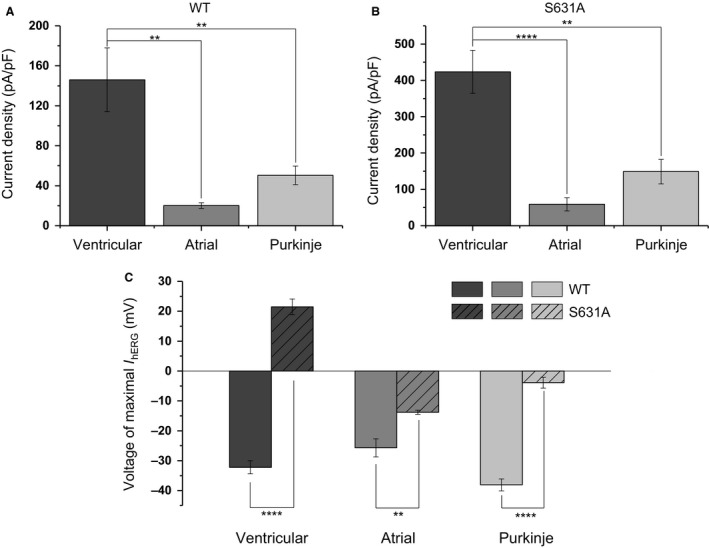
Peak *I*_hERG_ density during AP clamp commands. (A) Maximal *I*_hERG_ density for WT hERG channels during ventricular, atrial, and Purkinje fiber AP commands (*n* = 6 for all protocols). (B) Maximal *I*_hERG_ density for S631A hERG channels during ventricular, atrial, and Purkinje fiber AP commands (*n* = 7, 6, and 6 cells, respectively). Significant differences at *P* < 0.01 and *P* < 0.0001 are signified by ** and ****, respectively. Note that the statistical comparisons shown in A and B were made between the different AP waveforms for each of WT and S631A hERG and not between the two constructs. (C) Plots of the mean voltage at which peak repolarizing *I*_hERG_ occurred during each of the 3 AP waveforms for WT and S631A *I*_hERG_. For the ventricular AP waveform, WT *n* = 6 and S631A *n* = 7. For the atrial AP and PF waveforms, both WT and S631A *n* = 6. Significant differences at *P* < 0.01 and *P* < 0.0001 are signified by ** and ****, respectively.

The summary bar charts in Figure 4 show the magnitude of maximal *I*_hERG_ (normalized to cell capacitance and expressed as current density) elicited by each of the three waveforms for WT (Fig. 4A) and S631A *I*_hERG_ (Fig. 4B). As in a previous study of N588K hERG (McPate et al. 2009), we have compared magnitudes of peak repolarizing current density between the different AP commands for each channel type. Thus, for WT *I*_hERG_, the magnitude of peak repolarizing current during the ventricular AP was ~7.3 fold that during the atrial AP and ~2.9 fold that during the PF AP. For S631A *I*_hERG_ the magnitude of peak repolarizing current during the ventricular AP was ~7.2 fold that during the atrial AP and ~2.8 fold that during the PF AP. The most notable comparison here is between ventricular and PF APs, as both cell types contribute to overall repolarization profile of the ventricles; our results indicate that the ratio between peak repolarizing *I*_hERG_ in ventricular versus PF APs was similar for S631A and WT *I*_hERG_. Figure 4C illustrates graphically the mean values for the voltages at which peak repolarizing current occurred for WT and S631A *I*_hERG_ for each of the three AP command waveforms, thereby demonstrating for each AP type the shift in the timing of maximal current to earlier (corresponding to a more positive voltage) during AP repolarization for S631A *I*_hERG._

### Effect of 1 *μ*mol/L quinidine under AP clamp

**Figure 5:**
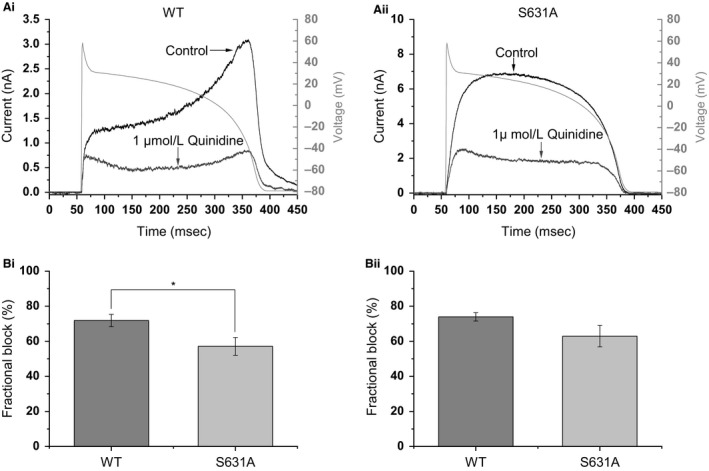
*I*_hERG_ response to quinidine. (A) Representative trace of WT (Ai) and S631A (Aii) *I*_hERG_ during ventricular AP in the absence (black) or presence (gray) of 1 *μ*mol/L quinidine. (Bi) Reduction in maximal WT (*n* = 5) and S631A (*n* = 6) *I*_hERG_ recorded during ventricular AP following quinidine application. Significant difference at *P* < 0.05 is signified by *. (Bii) Reduction in total charge (current integral) carried throughout the AP following application of quinidine. There was no statistically significant difference between WT (*n* = 5) and S631A (*n* = 6) *I*_hERG_ in the extent of quinidine reduction of the current integrals.

Prior experiments performed using conventional voltage clamp have shown that the inhibitory action of quinidine is comparatively little affected by attenuated inactivation hERG mutants (Lees‐Miller et al. 2000; Wolpert et al. 2005; McPate et al. 2008). In the final experiments of this study we tested a partial *I*_hERG_ blocking concentration of quinidine under AP clamp. Figure 5Ai and Aii show representative traces of *I*_hERG_ elicited by a ventricular AP command in control and following application of 1 *μ*mol/L quinidine. *I*_hERG_ magnitude was reduced by quinidine for both channels. Figure 5Bi shows mean fractional block of peak repolarizing current during the AP by 1 *μ*mol/L quinidine: for WT *I*_hERG_ the mean block was 71.9 ± 3.5% (*n* = 5), while for S631A hERG this was 57.1 ± 5.1% (*n* = 6; *P* < 0.05). We also quantified inhibition by measuring the reduction in total charge (current integral) carried by hERG throughout the AP and Figure 5Bii shows this information for WT and S631A *I*_hERG_ (mean fractional reductions of 74.0 ± 2.4% and 63.0 ± 6.2%, respectively; *P* > 0.05). Taken together, these data indicate that peak *I*_hERG_ block by quinidine was only modestly reduced by the S631A mutation, while the reduction in total charge carried by hERG during the AP command by quinidine was statistically similar between WT and S631A conditions.

## Discussion

### Historical context of the S631A hERG mutation

**Figure 6:**
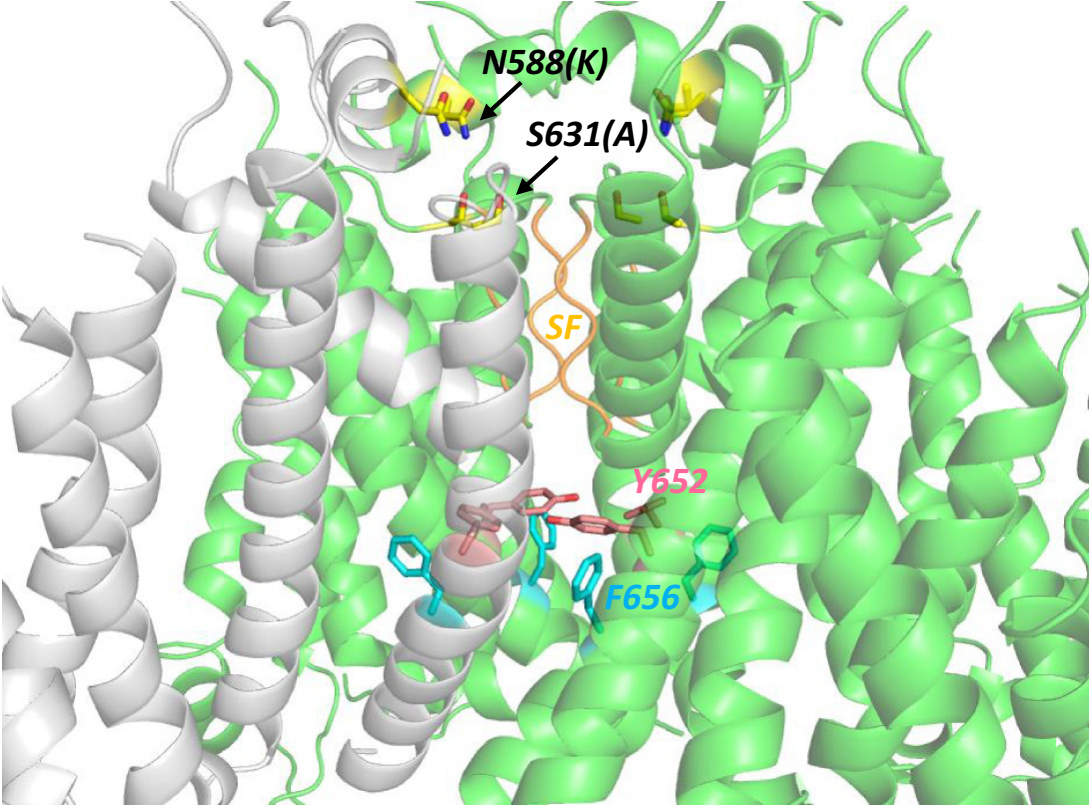
Location on hERG of the S631 residue. S631 and other key residues discussed in the text are highlighted in the open channel structure of a hERG construct obtained by cryo‐electron microscopy (PDB 5VA1; Wang and MacKinnon 2017). S631 lies adjacent to the top of the K^+^ selectivity filter (SF; S_624_VGFG_628_). N588 lies close to the top of the selectivity filter on a helical segment of the extracellular turret. S6 helix pore residues Y652 and F656 which are important for binding of quinidine to hERG are indicated and this region of the K^+^ permeation pathway below the selectivity filter is the likely binding site for quinidine. One of the four hERG subunits is colored gray.

The S631A hERG mutation is a notable example of an ion channel mutation found to be of clinical significance (Akdis et al. 2018) long after it had been made artificially to probe ion channel structure‐function. The S631 residue is located in the outer pore of the hERG channel, outside the selectivity filter (Fig. 6; (Wang and MacKinnon 2017)). In early work to establish the underlying basis of *I*_hERG_ inactivation, cytoplasmic N terminal deletion was found not to eliminate inactivation, whereas mutation of S631 to alanine (the homologous residue in EAG channels that lack the rapid inactivation of hERG channels) largely eliminated inactivation over physiologically relevant membrane potentials (Schoenherr and Heinemann 1996; Zou et al. 1998). Experiments utilizing *Xenopus* oocyte expression showed voltage‐dependent inactivation to be positively shifted by +102 mV, whereas voltage‐dependent activation was unaltered by the mutation (Zou et al. 1998). Work from this laboratory using a rabbit modified ventricular AP under AP clamp showed a substantially modified *I*_hERG_ profile during AP repolarization (Hancox et al. 1998b). Using mammalian cell expression and recording at physiological temperature we subsequently observed little alteration to *I*_hERG_ activation and a profound positive (~ +88 mV) shift in voltage‐dependent inactivation with S631A, effects that were similar to those of the N588K SQT1 mutation in the same study (McPate et al. 2008). On this basis, following the recent identification of the S631A mutation in an SQTS family (Akdis et al. 2018), we proposed that the clinical consequences of the S631A mutation, and potential pharmacological treatments, are likely to be similar to those of the spatially distinct N588K mutation (the location of which is shown in Figure 6 for comparison with S631) (Hancox and Stuart 2018). The results in this study bear out these predictions.

### Ventricular consequences of S631A *I*_hERG_ profile under AP clamp

The AP clamp method enables current measurement during dynamic, physiological waveforms and thus membrane potential “history” influences the current(s) of interest elicited under AP clamp (Hancox et al. 1998a; Noble et al. 1998; McPate et al. 2009). The data with human ventricular AP clamp in Figure 2 of this study show both augmentation in *I*_hERG_ magnitude due to the S631A mutation and a positive shift in peak repolarizing current, with instantaneous *I*–*V* and *G*–*V* relations similar to those reported previously for the N588K mutation (Cordeiro et al. 2005; McPate et al. 2005, 2009). While S631A *I*_hERG_ amplitude was significantly augmented during the AP, direct comparison between WT and S631A current amplitudes here is precluded by the necessity to use less of the S631A hERG expression construct to avoid excessively large currents under voltage clamp. However, prior simulation data have shown that the kinetic changes due to the N588K mutation are able to increase I_Kr_ amplitude to ~5‐fold that for the WT condition (Adeniran et al. 2011), whereas myocyte data with the hERG activator ICA‐105574, which profoundly positive‐shifts *I*_hERG_/*I*_Kr_ inactivation, have shown even larger proportionate increases in current amplitude (Gerlach et al. 2010). Thus, attenuation of *I*_hERG_ inactivation by S631A can be expected to lead to profound augmentation of *I*_hERG_. At the single channel level, the S631A mutation has also been reported to increase ion translocation rate (Zou et al. 1998), which would be anticipated also to contribute to increased macroscopic *I*_hERG_/*I*_Kr_.

Similar to prior studies of N588K hERG (Brugada et al. 2004; Cordeiro et al. 2005; McPate et al. 2005, 2009) we compared the properties of singly expressed WT and S631A mutant channels under AP clamp, while the index patient was heterozygous for the S631A mutation. In this regard, it is notable that recent work using concatenated hERG tetramers containing variable numbers of subunits with the S631A mutation has shown that while the effect of S631A in terms of inactivation is graded, it is not linearly so (Wu et al. 2014). Thus, in comparison to a concatemer with four WT subunits, *I*_hERG_ inactivation *V*_0.5_ was shifted by +53, +56, +68, and +77 mV with 1, 2, 3, and 4 S631A‐containing subunits, respectively (Wu et al. 2014). This indicates that heterozygous S631A expression can produce >70% of the inactivation impairment of homozygous expression and the results shown here are therefore likely substantially to be relevant to the clinical situation. Furthermore, the close similarity of effects under ventricular AP clamp of the S631A and N588K mutations (Cordeiro et al. 2005; McPate et al. 2005, 2009) means that SQTS simulations that have been performed previously to mimic the N588K mutation at cell and tissue levels (Adeniran et al. 2011) are likely also to be relevant to the S631A mutation. Incorporation of the kinetic changes to I_Kr_ resulting from the N588K mutation into ventricular cell and tissue models led to abbreviation of ventricular AP duration and of effective refractory period and to augmentation of voltage heterogeneity in localized regions of ventricular tissue, leading to increased susceptibility to unidirectional conduction block and re‐entry (Adeniran et al. 2011). In 2D and 3D simulations, the N588K mutation led to a reduced substrate size necessary for re‐entry and thereby facilitate re‐entrant spiral and scroll waves (Adeniran et al. 2011). The S631A mutation can be anticipated to produce similar electrophysiological changes in the ventricular myocardium, and these may account for instances of arrhythmic syncope and sudden death in the affected family (Akdis et al. 2018).

### S631A *I*_hERG_ during atrial and Purkinje fiber APs

SQTS is characterized by atrial as well as ventricular arrhythmias (Maury et al. 2008; Harrell et al. 2015; Hancox et al. 2018) and SQT1 patients with the N588K mutation have been reported to exhibit atrial fibrillation (Brugada et al. 2004; Hong et al. 2005). In atrial simulations, incorporation of the N588K mutation led to abbreviation of the effective refractory period and reduction of the wavelength for re‐entry, facilitating induction of spiral waves (Loewe et al. 2014). As yet, there is no clinical evidence as to whether the S631A hERG mutation increases susceptibility to atrial fibrillation or tachycardia; the only clinical information currently available reports arrhythmic syncope and familial instances of sudden death (Akdis et al. 2018). The results of our atrial AP clamp experiments are of relevance in this regard, however, as they demonstrate increased *I*_hERG_ during AP repolarization with the S631A mutation. The lower AP plateau of the atrial AP command resulted in less extensive *I*_hERG_ activation and hence a smaller net current in both WT and mutant conditions than the corresponding condition during the ventricular AP command. However, there was a clear shift in the timing of peak *I*_hERG_ to earlier following the AP peak, along with augmentation in current. This can be predicted to shorten atrial APD and effective refractory period and, thereby, to facilitate atrial re‐entry (Nof et al. 2010; Loewe et al. 2014). Clinical verification of such changes would require electrophysiological measurements of atrial refractoriness from individuals harboring the S631A mutation.

Our data also show a significant alteration in the *I*_hERG_ profile during the Purkinje fiber AP waveform, consistent with accelerated repolarization in the PF system also. Differences between PF and ventricular APs result in different extents of *I*_hERG_ activation and current magnitude and this is evident in both WT and S631A conditions. The extent of the difference was similar under S631A and WT conditions indicating that, under the conditions of this study at least, the S631A mutation did not exacerbate ventricular–PF differences in current magnitude, though the mutation differentially affected the timing of peak current during the two waveforms (Fig. 4C). The possible contribution to ventricular arrhythmogenesis (syncope/sudden death; (Akdis et al. 2018)) of ventricular‐PF differences in effects of the S631A mutation are not known. Prior simulations of ventricular arrhythmogenesis with the N588K mutation showed that changes to the electrophysiological properties of the ventricular myocardium with that mutation sufficed to produce an arrhythmogenic substrate without incorporation into the model of a PF network (Adeniran et al. 2011). However, incorporation of PFs in future simulations (for both the N588K and S631A mutations) would be valuable, in order to determine how changes to PF activity contribute to the overall ventricular arrhythmogenic substrate.

### Effectiveness of quinidine

While many SQTS patients, including the index patient with the S631A mutation, have received implantable defibrillators to protect from sudden death (Maury et al. 2008; Akdis et al. 2018; Hancox et al. 2018), adjunct pharmacology is also desirable to offset abbreviated repolarization and decrease arrhythmia risk (Hancox et al. 2018). Prior studies using conventional voltage clamp have already shown that, despite interacting with canonical drug binding aromatic residues on the S6 helices (Y652 and F656 (Lees‐Miller et al. 2000; Sanchez‐Chapula et al. 2003) the positions of which are shown in Fig. 6), in comparison with Class III methanesulfonanilide agents, quinidine is relatively insensitive to perturbation of the hERG channel's inactivation process (Lees‐Miller et al. 2000; Wolpert et al. 2005; McPate et al. 2006, 2008; Perrin et al. 2008). Thus, under conventional voltage clamp, the IC_50_ for S631A *I*_hERG_ inhibition by quinidine has been reported to be ~1.25‐fold to 3.5‐fold that of WT *I*_hERG_ (Lees‐Miller et al. 2000; McPate et al. 2008). The present data using 1 *μ*mol/L quinidine under AP clamp are consistent with this, showing only a modest decrease in the extent of S631A channel inhibition compared to the WT channel when effects on peak *I*_hERG_ during repolarization were assessed and no statistically significant decrease in quinidine reduction of the *I*_hERG_ integral during the ventricular AP. The N588K SQT1 hERG mutation has previously been demonstrated to have quantitatively similar effects on potency of quinidine inhibition of *I*_hERG_ under conventional voltage clamp (with IC_50_ values 3.5‐ to 5.8‐fold those for WT *I*_hERG_; (Wolpert et al. 2005; McPate et al. 2008)) and (hydro)quinidine is effective in prolonging the QT interval in that form of SQT1 (Wolpert et al. 2005; Giustetto et al. 2011; Villafane et al. 2013). Importantly, (hydro)quinidine has recently also been confirmed to be associated with prevention of life‐threatening arrhythmic events in SQTS patients (Mazzanti et al. 2017). For the N558K mutation at least, simulation data suggest that the beneficial actions of quinidine on effective refractory period result from both *I*_hERG_/*I*_Kr_ inhibition and the drug's Na channel inhibitory action (Whittaker et al. 2017). At present there are no patient data regarding effectiveness of quinidine in S631A hERG linked SQT1 (Akdis et al. 2018). However, the similarity between the effects of the N588K and S631A mutations on potency of *I*_hERG_ inhibition by quinidine in a direct comparison (McPate et al. 2008) and the known effectiveness of the drug in N588K hERG linked SQT1 strongly suggest that quinidine is likely to be effective at delaying repolarization in the setting of S631A‐linked SQT1. Issues with (hydro)quinidine availability in some countries (Mazzanti et al. 2017) may be problematic for its use, however. In relation to this, it is worth highlighting that conventional voltage clamp experiments have shown that the Class Ia agent disopyramide also retains effectiveness against the S631A mutation (Paul et al. 2001; McPate et al. 2008) and so disopyramide might provide a viable alternative where quinidine is unavailable.

## Conclusion

This study has demonstrated marked alterations in *I*_hERG_ profile during human atrial, ventricular, and Purkinje fiber AP waveforms with the S631A SQT1 mutation, which would be anticipated to accelerate AP repolarization and abbreviate refractoriness. Quinidine retains effectiveness against S631A hERG under ventricular AP clamp and this provides a rational basis for its deployment in this form of the SQTS.

## Conflicts of Interest

None.
